# Mechanobiology of the nucleus during the G2-M transition

**DOI:** 10.1080/19491034.2024.2330947

**Published:** 2024-03-27

**Authors:** Joana T. Lima, Jorge G. Ferreira

**Affiliations:** a Epithelial Polarity and Cell Division Laboratory, Instituto de Investigação e Inovação em Saúde (i3S), Porto, Portugal; b Departamento de Biomedicina, Unidade de Biologia Experimental, Faculdade de Medicina do Porto, Porto, Portugal; c Programa Doutoral em Biomedicina, Faculdade de Medicina, Universidade do Porto, Porto, Portugal

**Keywords:** Chromosome, G2-M transition, LINC complex, mechanotransduction, nuclear envelope, nuclear pore complex, nucleus

## Abstract

Cellular behavior is continuously influenced by mechanical forces. These forces span the cytoskeleton and reach the nucleus, where they trigger mechanotransduction pathways that regulate downstream biochemical events. Therefore, the nucleus has emerged as a regulator of cellular response to mechanical stimuli. Cell cycle progression is regulated by cyclin-CDK complexes. Recent studies demonstrated these biochemical pathways are influenced by mechanical signals, highlighting the interdependence of cellular mechanics and cell cycle regulation. In particular, the transition from G2 to mitosis (G2-M) shows significant changes in nuclear structure and organization, ranging from nuclear pore complex (NPC) and nuclear lamina disassembly to chromosome condensation. The remodeling of these mechanically active nuclear components indicates that mitotic entry is particularly sensitive to forces. Here, we address how mechanical forces crosstalk with the nucleus to determine the timing and efficiency of the G2-M transition. Finally, we discuss how the deregulation of nuclear mechanics has consequences for mitosis.

## Introduction

In addition to the biochemical signals that arise from the microenvironment, cells must be able to sense and integrate mechanical inputs to regulate proliferation and maintain homeostasis. Therefore, mechanotransduction, the conversion of mechanical forces into biochemically relevant information, contributes to numerous physiological and pathological processes such as embryonic development, muscular dystrophies, and cancer [1,2]. Mechanical responses are often mediated by load-bearing subcellular structures, such as the plasma membrane, cell adhesion complexes, and the cytoskeleton [3,4]. However, in recent years, the nucleus has emerged as a master regulator of cellular mechanical responses [5]. Indeed, dynamic changes in nuclear components can modify the mechanical properties of the nucleus, affecting its structural arrangement, chromatin anchoring, 3D chromosome conformation, and gene expression [6].

Many cellular events, including the cell cycle, are influenced by mechanical force. By temporally controlling mechanical signals, cell–cell- and cell–substrate-generated forces can regulate cell cycle transitions and their overall duration [7–11]. Accordingly, modifying substrate rigidity promotes the transition from the G1 to S phase, changing cell proliferation rates [11]. In addition, prolonged cell–cell tension and cell stretching can accelerate the transition from G2 to mitosis (G2-M) by triggering degradation of the CDK1 inhibitor Wee1 [9] and activating cyclin B1 transcription [8]. Mechanical cues can also affect multiple aspects of cell division, including mitotic duration [12], the efficiency of spindle assembly [13], and chromosome segregation [10], as well as cytokinesis [14]. Interestingly, the nucleus and some of its components are central to many of these mechanical responses. Accordingly, nuclear tension can stimulate transport across the nuclear envelope (NE) [10,15], possibly by inducing conformational changes in nuclear pore complexes (NPCs) [16]. Furthermore, these cell cycle changes are often accompanied by nuclear and chromatin rearrangements [17], which are reflected in modifications of the transcriptional landscape involving multiple pathways such as the Hippo pathway and its yes-associated protein (YAP) [18], focal adhesion kinase (FAK) signaling [19], and the Wnt pathway (through β-catenin) [20].

Although significant progress has been made toward clarifying the role of the nucleus in the generation and transduction of mechanical signals, a complete picture is still lacking. This is especially true when it comes to understanding how the extensive nuclear rearrangement that occurs during preparation for mitosis can influence its mechanical properties, as most of the direct evidence available today comes from studies performed in interphase cells. Here, we provide an overview of the key findings and current understanding of the events that promote the reshaping of the nuclear structure and mechanics during mitotic entry and their consequences for later stages of cell division.

## The nucleus during mitotic entry

**Figure 1.**
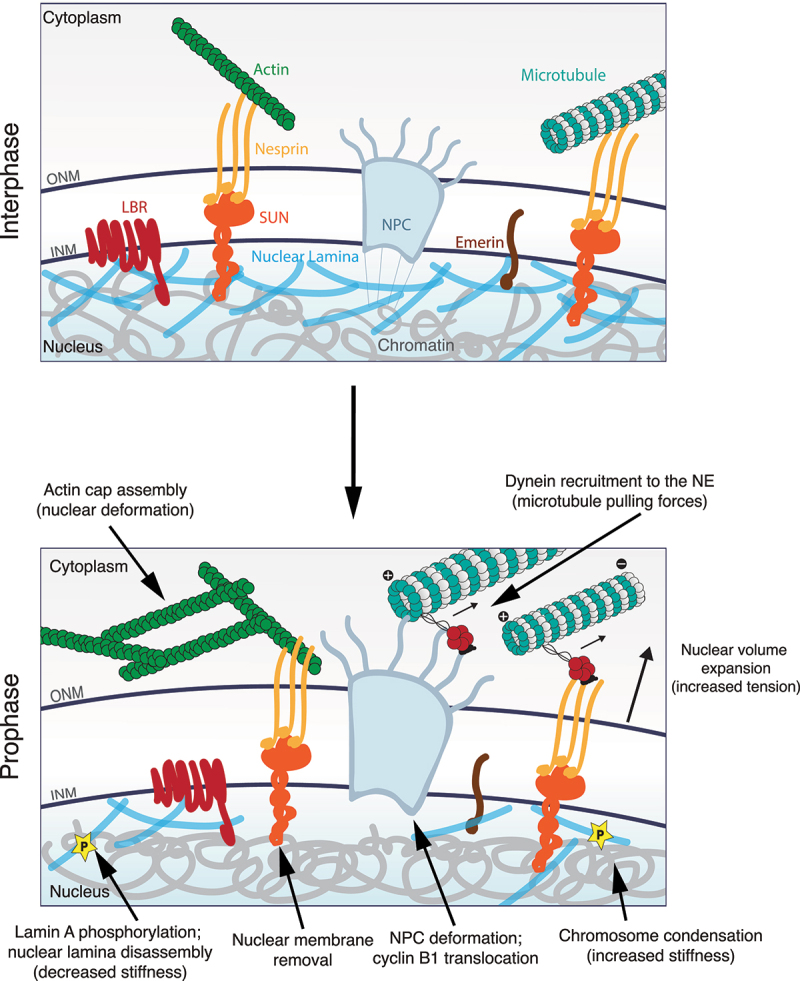
Structure of the nuclear envelope (NE) and its main mechanically regulated components.

In eukaryotic cells, the nucleus acts as a physical barrier that separates chromosomes from the cytoplasm (Figure 1). During cell division, these chromosomes must be correctly partitioned to ensure the formation of two genetically identical daughter cells from a single mother cell. This is achieved through a series of highly regulated events in a process known as mitosis. Errors in this process often lead to imbalances in chromosome copy number and can result in genetic instability (for a review, see [21]). Owing to the presence of the nuclear envelope (NE), which acts as a physical barrier, eukaryotic cells have evolved several strategies to ensure the efficiency of chromosome segregation. This has given rise to the terms ‘open’ and ‘closed’ mitosis, depending on whether cells disassemble their nucleus or not, to segregate their chromosomes. Here, we do not focus on the different modes of nuclear envelope remodeling during mitosis, which was covered in a recent review [22]. Instead, we will focus on the nuclear changes that occur when cells disassemble their NE in preparation for ‘open’ mitosis and the impact of mechanical forces on this process.

Nuclear pore complexes (NPCs) are embedded in the NE at the fusion sites of the inner and outer membranes (INM and ONM, respectively). The INM proteome contains a variety of transmembrane domain-containing proteins, such as LBR, the LEM domain containing Emerin, and SUN proteins. Notably, these SUN proteins bind to nesprins that localize to the ONM, across the perinuclear space. Nesprins can also bind to a variety of cytoskeletal elements, such as microtubules and actin, whereas SUNs bind to the nuclear lamina and chromatin in the nuclear interior. This physical link generates a mechanically competent connection across the NE, which allows for efficient force transmission. In the nuclear interior, the nuclear lamina lines the INM, acting as a shock absorber and helping the cell to modulate its response and adapt to mechanical stress. Moreover, the nuclear lamina interacts with chromatin within specific domains (lamina-associated domains, LADs) that regulate gene expression. Many of these components are dynamically regulated during the G2-M transition, triggering changes in nuclear structure during the prophase.

During the G2-M transition, cells are subjected to multiple external and internal forces that can affect the timing of mitosis. In line with this, prolonged cellular tension accelerates mitotic entry by upregulating cyclin B1 expression and activity [8,9]. Similarly, acute force application during late G2 triggers fast cyclin B1 nuclear translocation and mitotic entry [10], whereas reducing substrate stiffness has an opposite effect [10]. Overall, these can affect the outcome of mitosis [9,10,23,24]. Although the impact of extracellular forces on mitotic cell rounding and metaphase spindle orientation have been extensively studied [24–29], the interplay between mechanical forces and the structural changes that occur in the nucleus during mitotic entry remains poorly characterized.

**Figure 2.**
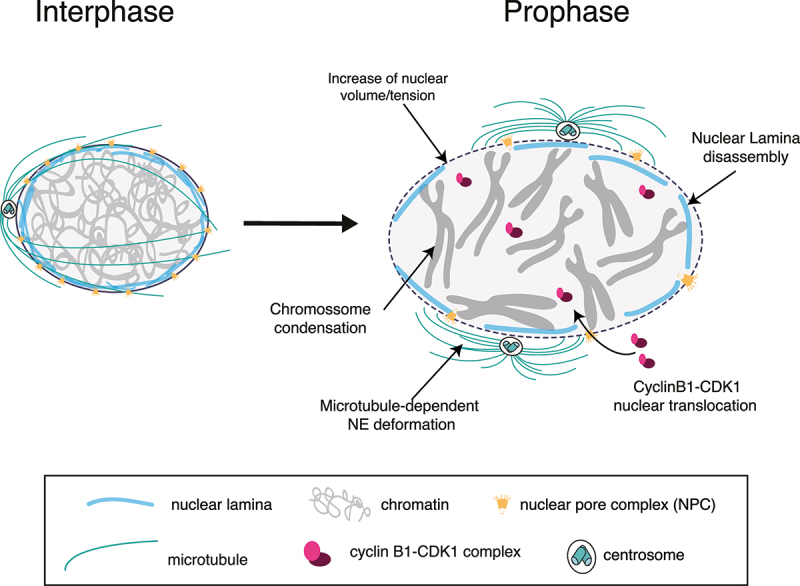
Mechanically induced changes on the nucleus as cells prepare for mitosis.

The first observable events in mitosis occur inside the nucleus, as cells condense their chromosomes into two sister chromatids during the early prophase [30,31] (Figure 2). This is accompanied by an increase in cell and nuclear volume [32,33] due to an influx of water, likely triggering an increase in nuclear tension [10]. Shortly thereafter, nuclear pore complexes (NPCs) and nuclear lamina disassemble, resulting in nuclear envelope breakdown (NEB). Importantly, chromatin [34,35] and nuclear lamina [35,36] have been shown to determine nuclear mechanics during interphase. Whether they play a role in shaping nuclear mechanics during the G2-M transition remains unclear.

During the G2-M transition, chromosomes condense, and nuclear volume and tension increase. This facilitates translocation of cyclin B1 across nuclear pore complexes (NPCs). This is followed by disassembly of the NPCs and nuclear lamina, which, in combination with forces generated by microtubules acting on the NE, results in nuclear envelope breakdown (NEB).

Many of the changes in nuclear structure that occur during the G2-M transition are controlled by mitotic kinases such as the cyclin B1-CDK1 complex and PLK1 [37–39]. These are responsible for the disassembly of NPCs [40] and nuclear lamina [41], which occur closer to NEB. To do so, these kinases must translocate to the nucleus. Consequently, the cyclin B1-CDK1 complex was proposed to coordinate cytoplasmic and nuclear events during the G2-M transition [38,42]. Notably, the nucleoplasmic accumulation of cyclin B1 was recently shown to depend on nuclear tension, suggesting that mitotic entry could be mechanically regulated [10]. How the nucleus senses these mechanical signals to control the G2-M transition is an interesting question for future research.

## The nuclear envelope

The nucleus and its contents are encompassed by a double-membrane structure, termed the nuclear envelope (NE), which is responsible for maintaining the composition of the nuclear space distinct from the rest of the cell (Figure 1). It acts as a barrier for passive macromolecule diffusion, protecting the genome against cytoplasmic proteins [43], ensuring the accurate control of gene expression [44], and is also involved in lipid biosynthesis and metabolism [45]. The NE consists of two distinct lipid bilayers, known as the inner nuclear membrane (INM) and the outer nuclear membrane (ONM), which are separated by a ~ 30–50 nm-wide perinuclear space [46,47]. The two NE membranes are fused in hundreds of discrete sections, where nuclear pore complexes (NPCs) are inserted. The ONM is contiguous with the rough endoplasmic reticulum (ER), which helps create different protein compositions between the INM and ONM [46]. Moreover, even though there is a continuity of the lipid membrane between the ONM and ER, they also have diverse resident proteins [46], which contributes to their different biological functions.

Interestingly, the NE is emerging as an important regulator of cellular mechanical responses. Although direct measurements of the NE tension have not yet been performed, recent studies have shown that during nuclear swelling or upon compression, the NE acts as a large surface reservoir, which is essential for accommodating the changes in nuclear shape imposed by these mechanical stimuli [48–50]. Accordingly, the nuclear surface of isolated HeLa cell nuclei can increase by up to 60% upon swelling, without rupturing. This increase in the nuclear surface triggers the recruitment and subsequent activation of cytosolic phospholipase A2 (cPLA2) [51] to the INM [49,50]. This leads to actomyosin activation and an upregulation of cellular contractility, making it an important player in the response to physical constraints. Importantly, this tension-sensing mechanism also acts during the G2-M transition, to regulate the timing of mitotic entry. As cells prepare to enter mitosis, nuclear area increases and cPLA2 is recruited to INM [10], facilitating the translocation of cyclin B1 across the NPCs. However, the molecular trigger for this nuclear expansion during the G2-M transition remains unclear.

Forces generated on the NE are also essential for the efficiency of NEB and early mitotic spindle assembly. During the prophase, the molecular motor dynein is recruited to NPCs [13,52–54] where it generates microtubule-mediated forces that pull on NE membranes. These forces are sufficient to generate the first visible holes on nuclear membranes [55,56] which, together with NPC disassembly [40], lead to nuclear permeabilization. In addition, these dynein-mediated forces are also essential for removing nuclear membranes from chromosomes, allowing their efficient capture by microtubules during early spindle assembly [57]. It is also worth noting that in many pathological conditions in which NE components are impaired, stretching of the nuclear membrane can lead to rupture. For example, during confined cell migration, increased nuclear pressure leads to the formation of nuclear blebs, which eventually result in nuclear rupture [58,59]. Similar ruptures of the NE also occur during the G2-M transition when nuclei are compressed [10], triggering premature mitotic entry that results in chromosome segregation errors. Overall, these reports highlight the importance of having a competent and mechanically stable NE that can absorb and react to changes in mechanics in and around the cell. However, a spatiotemporal characterization of the forces acting on the NE during the G2-M transition and how they help overcome the mechanical resistance of the nucleus is still lacking.

## LINC complex

One of the components of the NE directly linked to force transmission is the Linker of Nucleoskeleton and Cytoskeleton (LINC) complex. This protein complex is composed of SUN (Sad1 and UNC-84) proteins, which localize to the INM and extend their C-terminal domains into the perinuclear space. There, they interact with the C-termini of KASH (Klarsicht, ANC-1, and Syne Homology) domain-containing proteins, which reside in the ONM. Through their N-terminus, SUN proteins bind to the nuclear lamina [60], NPCs [61], chromatin [57,62] or other INM proteins, such as Emerin [63]. In contrast, nesprins (the KASH-containing proteins in mammalian cells) bind to different cytoskeletal elements, namely actin, microtubules, and intermediate filaments, also through their N-terminal domains [64,65]. There are four main forms of nesprins (nesprin 1–4) and two SUN proteins (SUN1 and SUN2) in mammalian cells. Due to their localization and interaction, these proteins act as a bridge between the nucleus and the cytoskeleton. This allows them to transfer tensile and shear forces across the whole cell, making them essential components of the mechanotransduction machinery [5,66,67]. Typically, LINC complexes assemble as trimers in a (3:3) configuration [46]. However, it has recently been shown that they are also capable of assembling higher-order structures (that is, 6:6 with different combinations of each protein) [68,69]. Importantly, it was proposed that the degree and efficiency of force transmission relayed by this complex depend on the type of structure formed [68].

In addition to these force-transmission properties, the LINC complex also plays a role in regulating cytoskeletal organization [66]. Through specific nesprins, the LINC complex interacts with different cytoskeletal components and their adaptors. For instance, by binding to microtubule-associated dynein [70], it can drive a variety of cellular functions, ranging from nuclear migration and organelle movement to NE-centrosome tethering [71–74]. In addition, the LINC complex can also bind to an ‘actin cap’, which consists of F-actin stress fibers that are formed over the nucleus during mechanical stimulation. This actin cap is capable of regulating nuclear morphology and motility [75–77]. Moreover, by modulating cell shape, it is possible to increase the recruitment of nesprins to this actin cap, further showing that the NE is not only a passive element in the transmission of mechanical force to the nucleus but also an active regulator of the process [78].

The LINC complex is also involved in several steps during the G2-M transition, from NE rupture to spindle assembly. During the prophase, through dynein-mediated microtubule interactions, the LINC complex ensures that centrosomes are tethered to the nucleus [79], allowing them to create invaginations on the NE, promoting fenestration of the nuclear membrane, and thus facilitating NEB [55,56,80]. Following NEB, the LINC complex contributes to spindle assembly in two ways. It is involved in the generation of an actomyosin network that clusters chromosomes around the mitotic spindle to facilitate their capture and congression [79,81] and also assists in removing NE remnants from chromosomes [62]. Accordingly, depleting SUN proteins leads to a significant delay in spindle assembly, possibly by impairing the interaction of microtubules with kinetochores. SUN2 has also been implicated in astral microtubule nucleation and metaphase spindle organization [12]. Therefore, depletion of SUN2 resulted in a delay in anaphase onset, which recapitulated the behavior of cells seeded on soft substrates. Interestingly, this delay seems to be dependent on Spindle Assembly Checkpoint (SAC) activity, as depleting SUN2 leads to increased levels of Mad2 on kinetochores [12]. Overall, these observations suggest that LINC complex-mediated force transmission is important for accurate spindle assembly. Notably, recent work done in the moss *Physcomitrium patens* demonstrated that SUN2 is required for timely chromosome alignment in metaphase. Here, the authors propose a model in which SUN2 facilitates the attachment of microtubules to chromosomes during spindle assembly by localizing them to the NE [82]. In combination, these studies demonstrate the importance of the LINC complex, not only during early spindle assembly but also at later stages of mitosis, due to its interaction with the actin and microtubule networks.

## Inner nuclear membrane (INM)

Other resident proteins of the INM can equally impact nuclear mechanics. For instance, emerin, a LEM domain-containing protein that binds to A-type lamins and chromatin [83] is phosphorylated in response to force [84,85]. Moreover, in combination with an intact nuclear lamina, emerin contributes to the rapid stiffening of nuclei when a force is applied to Nesprin-1 using magnetic tweezers [84]. Consequently, it is not surprising that emerin-deficient nuclei show altered NE elastic properties and decreased stability when force is applied [86]. Interestingly, when overexpressed, emerin localizes to the mitotic spindle and centrosomes and is required for NE reformation (NER) [87]. Further studies have shown that emerin deletion mutants inhibit nuclear reassembly *in vitro*, chromatin decondensation, and NPC reassembly [88] and can lead to severe chromosome segregation defects in *C.elegans* [89]. Similarly, the emerin interactor barrier-to-autointegration factor (BAF) is required to segregate and enclose chromosomes within the NE [90]. In *Drosophila*, BAF disruption can also prevent PP2A inactivation during mitosis, leading to persistent association with chromatin and a delay in anaphase onset and NE defects [91]. Furthermore, BAF, in conjunction with Emerin and MAN-1 (another LEM-domain-containing INM protein), has been implicated in the assembly of the nuclear lamina in *C.elegans* [92]. LAP2, another integral INM protein, has also been associated with cell cycle control. When mutated in HeLa cells, LAP2 inhibits the increase in nuclear volume normally observed during cell cycle progression [93]. In addition, knockdown of one of its isoforms (LAP2β) leads to misshapen nuclei, abnormal chromatin structure, and mislocalization of nuclear lamina components and nucleoporins, leading to cell death [94]. Similarly, knockdown of the Lamin B receptor (LBR), an additional INM protein, can result in nuclear aberrations such as nuclear blebs and micronuclei [95]. Conversely, overexpression of LBR is known to induce over-production of nuclear membranes [49], which decreases NE tension and cPLA2 recruitment to the NE during the G2-M transition [10]. Overall, these observations highlight the importance of INM proteins and their nuclear interactors in maintaining nuclear architecture and integrity, as well as ensuring an error-free cell division.

## Nuclear pore complex (NPC)

As previously mentioned, the two NE membranes fuse at discrete sites where the NPCs are inserted. These are the main regulators of nuclear-cytoplasmic transport, as they limit the transport of macromolecules larger than 50 kDa, while allowing the passage of smaller solutes such as ions, peptides, and amino acids, which can enter or exit the nucleus by passive diffusion [46]. NPCs cover approximately 11% of the nuclear surface area of normal HeLa cells [96]. Interestingly, NPCs were recently shown to be mechanosensitive. When force is applied, either externally or due to internal forces, nuclear membranes stretch, resulting in a dilated conformation of the NPCs [16]. This mechanically induced increase in NPC diameter could account for one-sixth of the total increase in nuclear membrane surface area in HeLa cells during nuclear swelling [97]. Importantly, when force is applied to the nucleus due to cell spreading, stretched NPCs can become more permissive to the passage of certain proteins such as YAP [15], altering the transcriptional program of these cells [11]. Similarly, recent work has shown that cyclin B1 translocation across NPCs during the G2-M transition is sensitive to the tension imposed on the nucleus [10]. This work also shows how premature import of cyclin B1 into the nucleus, triggered by cell confinement, can lead to chromosome segregation defects. However, this accelerated translocation of proteins across NPCs does not seem to be a universal feature for all forms of nuclear stretch. Indeed, osmotic swelling does not lead to an increased YAP translocation [15], whereas it accelerates cyclin B1 transport [10]. One possible but untested explanation for this difference in behavior could be a change in NPC structure during the G2-M transition, which would make them more permissive to force-induced transport. Nevertheless, the contribution of NPCs and the nuclear import/export machinery in response to the forces applied to the nucleus seems to significantly affect mitotic progression.

In addition to the regulation of nuclear transport, NPCs also provide a scaffold for centrosome anchoring during prophase. For instance, the cytoplasmic filament NPC component Nup358/RanBP2 helps in centrosome anchoring through its association with the adaptor protein Bicaudal D2 (BicD2), which can interact with molecular motor dynein, specifically during late G2 [52,98,99]. Other more centrally located NPC components, such as Nup133, can also provide anchoring to the dynein/dynactin complex, independently of the Nup358-BicD2 pathway [53]. Notably, depleting these NPC components blocks the recruitment of dynein to the NE during late G2/early prophase. In turn, this decreases microtubule pulling forces on NE membranes, leading to centrosome detachment and impaired nuclear membrane removal [53,62,100]. In addition, knockdown of the transport channel element Nup62 leads to defective centrosome separation, centriole maturation, and spindle orientation defects, which in turn lead to mitotic arrest and cell death [101].

NPC disassembly is also a decisive step in NEB, as it corresponds to the moment when the nucleus is permeabilized. This process is driven by the phosphorylation of many of its nucleoporin subunits (Nups), breaking protein – protein interactions at key structural contact points within the NPC [102,103]. This phosphorylation leads to the dispersal of NPC building blocks into the cytosol until nuclear envelope reformation (NER) after mitosis [104]. Although not yet tested, it is possible that forces applied to the nucleus affect the kinetics of NPC disassembly, given their role in mechanosensing and nuclear mechanotransduction [10,15,105,106]. Defining how forces applied to the nucleus affect NPC disassembly and the efficiency of NEB will be an important avenue of research in the future.

## Nuclear lamina

The nuclear lamina is a filamentous structure that lies underneath the INM and is comprised of intermediate filament proteins termed lamins that provide structural and mechanical stability to the nucleus [107,108]. There are two types of lamins: A-type lamins, comprising lamin-A and -C, and B-type lamins, comprising lamin-B1 and lamin-B2 [109,110]. Lamins interact with several INM proteins (such as emerin and SUN) and chromatin, either directly or indirectly, through chromatin-binding factors [111,112]. These lamin-associated domains or LADs are heavily enriched in transcriptionally repressed heterochromatin and have often been linked to chromatin organization and gene expression regulation [113]. Lamin loss has been linked to telomere hypermobility and transcription factor engagement by newly unrestricted chromatin, leading to changes in gene expression [114,115]. Importantly, the nuclear lamina plays a crucial role in determining how nuclei respond to mechanical forces by regulating nuclear shape, stiffness, and deformability under stress conditions. For instance, upon nuclear deformation, either by stretching or compression, the nuclear lamina acts as a ‘molecular shock absorber’, redistributing the force and protecting the NE from rupture [116]. This effect is mostly due to lamin-A; therefore, nuclei with lower levels of this lamin tend to be softer, as well as more deformable and fragile [107,117]. For this reason, the membranes of nuclei with lower levels of lamin-A are less capable of resisting compressive forces than their more rigid counterparts [97], making them more prone to rupture [117,118]. In contrast, cells expressing high levels of lamin-A and -C have higher nuclear stiffness, which hinders their ability to migrate through constrained environments [119]. Additionally, mechanical forces can also affect lamin behavior under normal conditions. Force application can lead to unfolding of lamins, changing the accessibility of epitopes [120]. Consequently, this decreases phosphorylation levels and stabilizes these filaments, possibly impairing their correct dissolution during mitotic entry. Therefore, placing cells on softer substrates results in lower cytoskeletal tension and triggers lamin-A/C phosphorylation, leading to increased mobility and turnover [121,122].

During mitotic entry and in parallel with NPC phosphorylation and disassembly, the nuclear lamina is solubilized by the action of mitotic kinases [41,123,124]. Considering their role in supporting nuclear mechanics, it is likely that the nuclear lamina can generate forces that work against NEB. Therefore, its disassembly should decrease the mechanical resistance [125] of the nucleus to microtubule-pulling forces [55,56] resulting in NE fenestration, ultimately facilitating NEB. In addition, Lamin A is involved in spindle assembly by targeting dynein to the cell cortex [126]. Interestingly, defects in lamin-A expression can induce chromosome segregation defects, such as anaphase DNA bridges [127], whereas defects in lamin-B have been linked to genomic instability [128,129]. It is possible that these changes are due to altered chromosome behavior, considering that lamin-deficient cells show altered chromosome distribution [130] and chromosome movement during meiosis [131]. Whether this holds true for mitotic cell division remains to be determined. Nevertheless, defects in lamin assembly and disassembly were correlated with high levels of aneuploidy, carcinogenesis, and aging [132–134].

Considering that the nuclear lamina directly interacts with NPCs [135–137], it is also crucial for NPC anchoring [138]. Indeed, the nuclear lamina helps counterbalance dynein pulling forces felt by NPCs, where dynein is anchored, thus contributing to efficient centrosome separation in prophase [139]. Therefore, in the absence of a mechanically stable nuclear lamina, NPCs tend to cluster around centrosomes due to dynein-mediated forces, impairing centrosome separation.

## Chromatin

The contribution of chromatin to nuclear mechanics has received considerable attention in recent years. Accordingly, chromatin is thought to modulate nuclear stiffness by controlling the response to small deformations [35]. Importantly, the ratio of euchromatin to heterochromatin in the nucleus seems to be a relevant contributor to nuclear stiffness. Indeed, nuclei with higher levels of heterochromatin are more rigid than those with decondensed chromatin [140–142]. Moreover, less compact chromatin is more mobile and deformable [140,143–145], which could account for decreased nuclear stiffness. Interestingly, this mechanical response of the nucleus seems to depend on chromatin interaction with INM proteins [146].

Importantly, chromatin organization and structure do not remain static throughout the cell cycle. It is widely known that during mitotic entry, chromatin must condense to form chromosomes, individual identities that can be easily segregated during later mitotic stages. Many proteins have been linked to chromosome condensation, such as condensins, topoisomerase IIα (TOPOIIα), and cohesins, whose depletion can lead to defects in mitotic chromosome formation and sister chromatid segregation errors [147–149]. Notably, chromosome condensation is also required for timely mitotic entry. This is supported by observations showing that the disruption of chromatin topology during G2 leads to a p38/MAPK checkpoint-dependent delay [150]. The initial steps of chromosome condensation occur during late G2/prophase under the regulation of the mitotic kinases CDK1 and Plk1 [151,152]. This process depends mostly on condensin II complexes [153] as well as histone post-translational modifications, such as histone H3 phosphorylation [154,155] and histone H4 deacetylation [156]. Importantly, condensins have previously been linked to the control of mitotic chromosome stiffness and stability by forming a scaffold for late-recruited proteins to enable the final steps of chromosome condensation and chromatid formation [34,157]. Moreover, condensed chromosomes display an increased stiffness when compared to interphase chromosomes [34,158,159]^,^, which also correlates with the higher nuclear tension observed during late G2/early mitosis [10]. Because this condensing chromatin is tethered to the NE at discrete sites, it can generate local inward pulling forces on the NE, which are sufficient to cause centripetal shape fluctuations [160]. Interestingly, impairment of chromatin condensation at the onset of mitosis due to treatment with trichostatin A (TSA), a pan-histone deacetylase inhibitor, greatly reduces these fluctuations, suggesting that chromosome condensation is an active mechanical component in this process. Therefore, it was proposed that chromosome condensation, in combination with forces generated by centrosome-nucleated microtubules, could weaken the NE membrane to facilitate NEB [56]. This would also explain why disrupting chromosome condensation induces a delay in the G2-M transition [150]. However, whether a causal relationship exists between chromosome condensation, nuclear tension, and the biochemical pathways that regulate the G2-M transition and NEB remains to be fully determined.

## Conclusions

The nucleus is a highly complex and dynamic structure (Figure 1) that governs multiple cellular processes. This dynamic nature is extremely evident during mitotic entry, when cells that undergo an ‘open’ mitosis must completely disassemble their nucleus in order to expose the newly condensed chromatin (now assembled into chromosomes) to microtubules emanating from the mitotic spindle, so that they can be correctly segregated into two equal daughter cells. During this brief period of the cell cycle, all nuclear components, such as nuclear membranes, NPCs, and the nuclear lamina must be dramatically reorganized. The disassembly of all these components, in collaboration with chromatin condensation, likely results in modifications of the mechanical properties of the nucleus [56,160], which ultimately culminates in its disassembly during NEB (Figure 2).

In this review, we demonstrate how different nuclear components contribute to the overall mechanical response of the nucleus. We provide evidence on how the nucleus responds to mechanical forces, either by modulating NPC diameter, altering nuclear import and transcriptional programs [15,17] or through the ability of the nuclear lamina in absorbing forces imposed by cell stretching and compression. Moreover, we explore how the nucleus itself can act as a source of mechanical cues not only in interphase through components such as the LINC complex [74] and LAP2 [93] but also during mitosis, as condensing chromosomes trigger changes in nuclear stiffness [144,146]. It is important to note that some of these changes are indirect, triggered by a cascade of downstream nuclear and cytoplasmic events. These cascades are often relayed by kinases and other molecular effectors, such as in the case of Lamin A phosphorylation following force application [122] or the E-cadherin mediated Wee1 degradation in low-confluency epithelia [9]. Nevertheless, we describe how multiple nuclear elements are linked to the G2-M transition, either by participating in NEB and early spindle assembly, or by ensuring accurate chromosome segregation [12,72,139,161,162]. These reports highlight how these factors contribute on multiple levels (from nuclear lamina structure to nucleoplasmic transport and nuclear membrane stability) to ensure timely mitotic entry and efficient spindle assembly.

Nevertheless, the precise nature of the mechanosensitive mechanisms underlying these processes and their respective implications for cell division are not yet fully understood. However, considering all the available evidence presented in this review, it is reasonable to assume that the nuclear ability to respond to forces changes during the G2-M transition. For instance, the depolymerization of the nuclear lamina that occurs during mitotic entry should alter nuclear stiffness. Similarly, during mitotic entry, dynein-mediated forces act on the NE to facilitate mitotic entry [55,56] and cooperate with SUN proteins in removing nuclear membranes from chromatin [62]. Interestingly, the LINC complex was shown to impact centrosome positioning during prophase, a process that is deregulated in some chromosomally unstable cell lines [72]. Nonetheless, direct measurements of nuclear stiffness, deformability, and tension during mitotic entry are still lacking and would be extremely informative. In addition, a detailed characterization of how forces acting on the nucleus during the G2-M transition contribute to the kinetics of NE disassembly and spindle assembly efficiency is still missing. This knowledge would allow us to understand how forces acting on the nucleus can impact later stages of mitosis and chromosome segregation efficiency, possibly paving the way for novel therapeutic strategies aimed at modulating the mechanical response of the nucleus during cell division.

## Data Availability

Data sharing is not applicable to this article, as no new data were created or analyzed in this study.
